# LncRNA TCONS_00023297 Regulates the Balance of Osteogenic and Adipogenic Differentiation in Bone Marrow Mesenchymal Stem Cells and the Coupling Process of Osteogenesis and Angiogenesis

**DOI:** 10.3389/fcell.2021.697858

**Published:** 2021-06-28

**Authors:** Haitao Wang, Peng Wei, Yi Zhang, Yuebai Li, Li Yin

**Affiliations:** ^1^Department of Orthopaedic Surgery, The First Affiliated Hospital of Zhengzhou University, Zhengzhou, China; ^2^Department of Gynaecology and Obstetrics, The First Affiliated Hospital of Zhengzhou University, Zhengzhou, China; ^3^Department of Biochemistry and Molecular Biology, School of Basic Medical Sciences of Zhengzhou University, Zhengzhou, China

**Keywords:** long noncoding RNA, bone marrow mesenchymal stem cells, osteogenesis, Adipogenesis, osteoporosis

## Abstract

Long noncoding RNA (lncRNA) is a noncoding RNA with a length of more than 200 bases. It plays an important role in the occurrence and development of diseases. Research on lncRNAs has received increasing attention. Bone is an important organ of the human body. As the population ages, the incidence of osteoporosis gradually increases. The mechanism of action of lncRNAs in the development of osteoporosis is unclear. The imbalance between osteogenic and adipogenic differentiation in bone marrow mesenchymal stem cells (hBMSCs) and the coupling process of osteogenesis and angiogenesis plays an important role in the development of osteoporosis. Therefore, this study focused on the mechanism by which lncRNAs regulate the osteogenic differentiation of bone marrow mesenchymal stem cells and the mechanism of action of lncRNAs in bone metabolism. The expression of lncRNAs in the osteogenic differentiation of hBMSCs was detected by lncRNA microarray. Real-time quantitative PCR was used to detect the expression changes of lncRNA and osteogenic genes during hBMSC osteogenic and adipogenic differentiation. The ceRNA mechanisms were detected by RIP and luciferase reporter gene assays. The effect of lncRNAs on the osteogenesis–angiogenesis coupling process was detected by Transwell assays. TCONS_00023297 increased expression during osteogenic differentiation; TCONS_00023297 overexpression promoted osteogenic differentiation of hBMSCs; BMP2 regulated TCONS_00023297 expression in a concentration- and time-dependent manner; TCONS_00023297 regulated miR-608 via a ceRNA mechanism; TCONS_00023297 inhibited hBMSC adipogenic differentiation; and TCONS_00023297 promoted VEGF secretion by hBMSCs. TCONS_00023297 regulates osteogenic differentiation, adipogenic differentiation, and osteogenic–angiogenic coupling of hBMSCs via the TCONS_00023297/miR-608/RUNX2/SHH signaling axis.

## Introduction

Since the establishment of the human genome project in 2003, it has been found that 70–90% of the genes in the genome can be transcribed, but only 1–2% of the genes have protein coding functions (Collins et al., 2003; Kapranov et al., 2007; Mercer et al., 2009; Green et al., 2015). These noncoding RNAs are also called noncoding RNAs (Meller et al., 2015). They lack conservative open reading frames and were once thought to have no biological regulatory function in cells, so they were also called “junk RNAs” (Wright and Bruford, 2011; Dhamija and Diederichs, 2016). However, in the past two decades, a large amount of evidence has shown that noncoding RNAs are not “garbage” of the genome. In contrast, they play irreplaceable role in the regulation of the cell phenotype. In terms of human skeletal muscle pathology, a long noncoding RNA (lncRNA) related to brachydactyly type E (BDE), a disease of palmoplantar shortening, has recently been found (Maass et al., 2012). Two autosomal dominant BDE families were identified to have translocation mutations on chromosome 12, which lead to genome disruption of CISTR-ACT, an important *cis* regulatory element. CISTR-ACT also produced lncRNA DA125942. It is clear that the CISTR-ACT site binds to the PTHLH site through *cis* action, which encodes parathyroid hormone-like hormone in the chondrogenesis regulator (Vortkamp et al., 1996); at the same time, CISTR-ACT interacts with Sox9 via trans action (Bi et al., 1999). Studies have revealed the important function of lncRNA-related HOX genes in the regulation of skeletal muscle development, and the HOX gene is a conserved family of developmental transcription factors that initiate specific processes during animal development (Pineault and Wellik, 2014). For example, lncRNAs produced by HOXA and HOXD clusters can regulate the growth and construction of limbs or the spine. Hotair was the first vertebrate lncRNA found to regulate the function of HOX (Salsi and Zappavigna, 2006). Hotair is expressed from the HoxC locus and recruits part of the EZH2, Suz12, and PRC2 complexes to bind to the Hoxd cluster. Thus, H3K27 methylation is used to establish a silent chromatin state, which results in inhibition of the HOXD gene. Knockout of hotair in mice leads to lumbosacral junction deformity and metacarpal and carpal deformity (Li et al., 2013).

Bone marrow mesenchymal stem cells have the ability to undergo multidirectional differentiation. As the common source of osteoblasts and adipocytes in bone marrow, in some physiological and pathological conditions, due to the increasing number of adipocytes in bone marrow, the microenvironment of the bone marrow cavity changes, which leads to an increase in BMSC differentiation into adipocytes and a decrease in osteogenic differentiation, thus breaking the balance between bone absorption and bone formation. The formation of a vicious cycle eventually leads to or aggravates osteoporosis (Georgiou et al., 2012; Ko et al., 2016). An *in vitro* cell culture study also confirmed that BMSCs from osteoporotic rats had a stronger adipogenic differentiation ability than BMSCs from normal rats; in contrast, the osteogenic differentiation ability was weakened. In patients with all kinds of primary osteoporosis, the adipogenic differentiation of BMSCs in the bone marrow cavity increases with decreasing osteogenic ability, and the content of adipocytes is inversely proportional to the degree of osteoporosis, which further indicates that there is “one ebb and another” relationship between adipocytes and osteoblasts in the bone marrow, which has been considered the pathogenesis of primary osteoporosis (Li et al., 2018; Muruganandan et al., 2020; Robert et al., 2020). Therefore, the new adipocytes formed by the directional differentiation of BMSCs have a close physiological and pathological correlation with fat and bone. The loss of bone mass in primary osteoporosis is the result of the correlation between fat and bone.

There have been many studies on the factors related to bone formation, among which the influencing factors of bone vessels have been a research hotspot in recent years. Under the condition of normal bone formation, mesenchymal stem cells continuously differentiate into osteoprogenitor cells and further form osteoblasts, thus participating in bone formation and maintaining bone homeostasis, which plays an indispensable role in the regeneration and repair of new bone formation and fracture healing; however, the entire process of bone formation requires vascular involvement. Therefore, the skeletal vascular system plays an important role in embryonic bone formation, adolescent bone growth, and subsequent bone remodeling (Ouyang et al., 2020). Recently, it has been found that the blood vessels of subtype H in the bone are accompanied by osteoprogenitor cells. The blood vessels of subtype H are strongly positive for endothelial cells (EPCs) and exist in the metaphysis and subperiosteum (Zhao and Xie, 2020). This kind of special blood vessel can regulate the growth of bone vascular tissue, produce special metabolic, and molecular microenvironments, maintain bone progenitor cells in peripheral blood vessels, and connect angiogenesis with bone formation. Therefore, it is of potential clinical significance to study the coupling between bone formation and blood vessels of the H subtype.

Therefore, this study focused on the mechanism by which lncRNAs regulate osteoporosis. We investigated the effect of lncRNAs on bone metabolism by studying the effect of lncRNAs on osteogenic and adipogenic differentiation of BMSCs and the coupling between osteogenesis and angiogenesis.

## Materials and Methods

### Isolation and Culture of Human Bone Marrow Mesenchymal Stem Cells

The specimens were collected from The First Affiliated Hospital of Zhengzhou University, which was approved by the ethics committee of the hospital, and the informed consent was obtained and signed. 10 ml of human bone marrow blood was added to Ficoll solution at a proportion of 1:1 in the lower layer of the centrifuge tube, and then, 10 ml of bone marrow blood was slowly added to the upper layer. The centrifugal speed was 2,000 rpm with up and down of 1 for 20 min. After centrifugation, the centrifuge tube was slowly removed. At this time, the liquid was stratified, and the mononuclear cell layer was carefully drawn into a new centrifuge tube. Then, 10 ml of PBS was added and centrifuged at 1,500 rpm for 10 min. One milliliter of culture medium (containing 1% penicillin Streptomycin Solution and 10% Australian fetal bovine serum within DMEM medium) was added to the suspension and inoculated into a 10 cm culture dish. The culture medium was changed every 3 days after inoculation. When the cells reached 80% healing, they were subcultured.

### LncRNA Microarray

Integrity and concentration of RNA were assessed after RNA extraction and prior to sample labeling. Arraystar Flash RNA Labeling Kit was used for sample labeling. Hybridization was performed in Agilent’s SureHyb Hybridization Chambers. After washing, slides were scanned with the Agilent DNA Microarray Scanner. Data was extracted using Agilent Feature Extraction software. Results were provided in the Raw Data Files. Normalization was performed using the Agilent GeneSpring GX v11.5 software. Further data analysis was performed using Agilent GeneSpring GX v11.5 software. Subgroup analysis was performed using home-made scripts.

### RNA Pull-Down Assay

RNA pull-down detection was performed according to previous research reports (Xu et al., 2015). Biotin RNA Labeling Mix (Roche Diagnostics) were used for RNA with biotin labeled and treated with RNase-free DNase I. The purified RNA were incubated with human bone marrow mesenchymal stem cells (hBMSCs) cell lysates at 25°C for 1 h. Complexes were isolated with streptavidin agarose beads (Invitrogen). The beads were washed briefly three times and boiled in sodium dodecyl sulfate buffer, and the retrieved protein was detected using the standard western blot technique. The RNA present in the pull-down material was detected using reverse-transcription polymerase chain reaction (RT-PCR).

### RT-qPCR Assay

Total RNA was extracted from hBMSCs cells by Trizol reagent, and RNA concentration was detected by NanoDrop. 1 μg RNA was reverse transcribed by RNA reverse transcription kit, RT-PCR was performed by SYBR Green RT qPCR Master Mix kit, and the sample was added according to the instructions of the kit. The reaction conditions are as follows: procedure 1: 95°C 30 s, 1 cycle; Program 2: 95°C 5 s, 50 cycles, 60°C 34 s; Program 3: 95°C 5 s 1 cycle, 65°C 60 s 97°C 1 s, program 4: 42°C 30 s 1 cycle. The relative gene expression was calculated by 2^–△^^△^^*Ct*^ method, and the mRNA expression was corrected by GAPDH expression.

### Luciferase Assay

Using Dual luciferase^®^ Reporter assay kit was used for detection. According to the experimental grouping, lipo2000 transfection kit was used for plasmid transfection. After 12 h of transfection, fresh medium was replaced. After 72 h of transfection, the cells were washed twice with PBS and 250 μl lysate was added into each well was used to lyse the cells. Add 100 μl of LAR II reagent into 96 well black plate, and then add 20 μl of lysate to detect the reading. 100 μl Stop & Glo substrate was added within 10 s to detect the reading.

### LncRNA Overexpression and Knockdown

LncRNA TCONS_00023297 was cloned into pLVX-IRES Puro vector and sequenced. Amplification of lncRNA TCONS_00023297 overexpression plasmid (pLVX-lncRNA TCONS_00023297) and the control plasmid (pLVX-vector) using DH5α competent cells. The plasmid was transfected into hBMSCs by Lipofectamine 2000. LncRNA TCONS_00023297 siRNA (sense sequence 5′-ACUAUGUGAAGUCAAACGGGA-3′, anti-sense 5′-CCGUUUGACUUCACAUAGUGA-3′) and control siRNA were purchased from GenePharma and dissolved in DEPC water at 20 μM. The transfection concentration was adjusted to 200 nM, and the siRNA was transfected into hBMSCs by Lipofectamine 2000.

### Alkaline Phosphatase Staining

On the seventh day of osteogenic differentiation induction of hBMSCs, the culture medium was poured out, and then, PBS was washed twice and fixed with 4% paraformaldehyde solution for 30 s. The fixative solution was aspirated and then washed twice with PBS. DMF was used to dissolve the substrate, and dye buffer was used to dissolve solid violet B. Then, the dissolved substrate and solid violet B were added into the buffer solution and fully mixed. The alkaline phosphatase (ALP) staining solution was added to the culture plate and incubated in a 37°C incubator for 45 min. After incubation, the plate was removed, and the ALP staining solution was removed. The PBS was washed twice, and scanning or microscopic observation was performed using a scanner.

### Alizarin Red Staining

On the 21st day of osteogenic differentiation induction of hBMSCs, the culture medium was poured out, PBS was washed twice, and then, a 4% paraformaldehyde solution was added for fixation for 20 min. After the paraformaldehyde solution was removed, it was washed twice with PBS. Alizarin red powder (1.3692 *g*) was added to 100 ml of PBS solution, and the pH value was adjusted to 4.1–4.3 with concentrated hydrochloric acid. The alizarin red staining solution was centrifuged at 2,000 rpm for 10 min. The supernatant was added to the culture plate and then placed on a shaking table. After dyeing for 20 min, the staining solution was removed and washed slowly with PBS. A scanner was used to scan or observe and take pictures under a microscope. We added 0.1M sodium hydroxide solution to the culture plate to dissolve the calcium nodules, the solution was added to a 96-well plate, and 100 μl of solution was added to each well. The absorption wavelength at 580 nm was detected by full wavelength enzyme-linked immunosorbent assay.

### Adipogenic Differentiation of Human Bone Marrow Mesenchymal Stem Cells

DMEM was supplemented with a 1% penicillin streptomycin solution, 10% fetal bovine serum (Australia origin), 1 μM dexamethasone, 500 μM 3-isobutyl-1-methylxanthine, 10 μg/ml insulin, and 60 μM indomethacin. HBMSCs were inoculated into the culture plate. When the hBMSCs grew to a 90% healing degree, adipogenic differentiation induction medium was added for 2 days, and then, adipogenic differentiation maintaining medium was added for 1 day. After 14 days of cyclic culture, oil red O staining was performed.

### Western Blot Analysis

The cell lysate was prepared according to the ratio of RIPA:PMSF = 100:1. Then, 120 μL of cell lysate was added into each well. The cells were scraped off the cell culture plate with a cell scraper and incubated on ice for 30 min. Centrifugation was performed at 12,000 rpm at 4°C for 15 min. Each sample was added to a 20 μg protein sample, and the electrophoresis conditions were adjusted to a constant pressure of 80 V. When the protein sample gathered at the junction of the concentrated gel and separation gel, the protein marker was separated, and the electrophoresis condition was changed to a constant pressure of 120 V. After activation, the PVDF membrane was placed in a transfer film solution, and the gel was removed. The film was transferred to a constant current of 300 mA. After the 90 min film was transferred, the PVDF membrane was removed. A 5% BSA solution was incubated for 1 h. The first antibody solution was added and incubated overnight at 4°C. The secondary antibody was incubated at room temperature for 1 h and then placed on a shaking table to prepare the developer. The TBST was sucked out, and the developer was added for development.

### Construction of a Heterotopic Osteogenesis Model

The hBMSCs were resuspended. The β-TCP material was spread on the bottom of a 24-well plate, and then, the material was soaked in culture medium. Then, 5 × 10^5^ cells were inoculated into each well. The plates were placed in a 37°C incubator and incubated for 12 h. The skin on both sides of the back and spine was cut, tricalcium phosphate carrier material loaded with the hBMSCs was inoculated into the subcutaneous muscular membrane, and the skin was sutured. After 2 months of ectopic implantation, the mice were sacrificed for cervical dislocation, and then, the specimens were removed.

### Statistical Analysis

Each experiment was verified three times, and all data are expressed as means ± standard deviations. The difference between the two groups was analyzed by two-tailed Student’s *t*-test. One-way ANOVA was used for statistical analysis of multiple groups of data. When *P* < 0.05, it was considered that there was a significant difference, ^∗^ means *P* < 0.05, ^∗∗^ means *P* < 0.01, and ^∗∗^ means *P* < 0.001.

## Results

### Expression of TCONS_00023297 Improved During Osteogenesis

**FIGURE 1:**
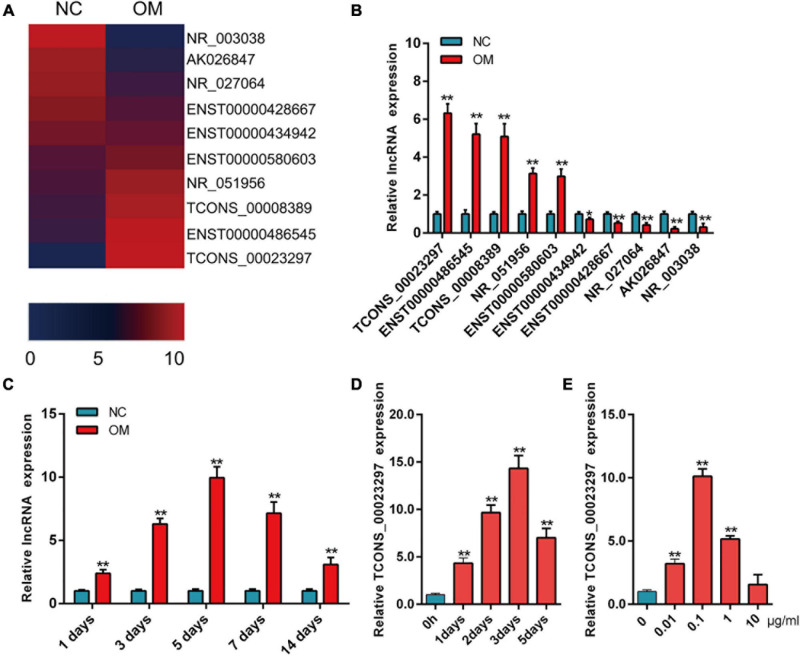
Expression of TCONS_00023297 improved during osteogenesis. **(A)** The expression of lncRNA was analyzed by hotmap, NC was indicated negative control and OM was indicated osteogenic medium. **(B)** Detection of lncRNA expression in osteogenic differentiation by qRT-PCR. **(C)** Expression of TCONS_00023297 in the osteogenic differentiation of hBMSCs detected by qRT-PCR. **(D)** Effect of BMP-2 on the expression of TCONS_00023297 at different time points. **(E)** Effects of different concentrations of BMP-2 on the expression of TCONS_00023297. All data are expressed as the means ± standard deviations. “*”means *P* < 0.05, and “**”means *P* < 0.01.

The results of the lncRNA microarray showed that lncRNAs were significantly up-regulated or down-regulated during osteogenic differentiation. We selected the lncRNAs that were significantly up-regulated and significantly down-regulated for hotmap analysis (Figure 1A). The expression of lncRNAs was detected by qRT-PCR on the third day of osteogenic differentiation. The qRT-PCR results showed that lncRNAs were significantly different on the third day of osteogenic differentiation, and the change trend was consistent with the results of lncRNA microarray detection (Figure 1B). TCONS_00023297 increased significantly during osteogenic differentiation (Figure 1B). The qRT-PCR results showed that the expression of TCONS_00023297 on the first, third, fifth, seventh, and 14th days of osteogenic differentiation was significantly higher than that of the control group and reached a peak on the fifth day (Figure 1C). The expression of TCONS_00023297 was detected by qRT-PCR on the first, second, third, and fifth days of osteogenic differentiation of hBMSCs induced by BMP-2 (0.1 μg/ml). The results showed that BMP-2 could significantly promote the expression of TCONS_00023297 on the first, second, third, and fifth days, and the expression of TCONS_00023297 was detected on the third day after induction and was the highest. These results suggest that BMP2 has a time-dependent effect on the expression of TCONS_00023297 (Figure 1D). qRT-PCR was used to detect the expression of TCONS_00023297 on the third day after BMP2 (0.01, 0.1, 1, and 10 μg/ml)-induced osteogenesis of hBMSCs. The results showed that different concentrations of BMP-2 had different effects on the expression of TCONS_00023297. BMP2 (0.01, 0.1, and 1 μg/ml) significantly promoted the expression of TCONS_00023297, 0.1 μg/ml BMP2 had the most obvious effect, and 10 μg/ml BMP2 had no effect on the expression of 00023297. The above results indicate that BMP2 has a concentration-dependent effect on the expression of TCONS_00023297 (Figure 1E).

### TCONS_00023297 Improved the Osteogenesis of the BMSCs

**FIGURE 2:**
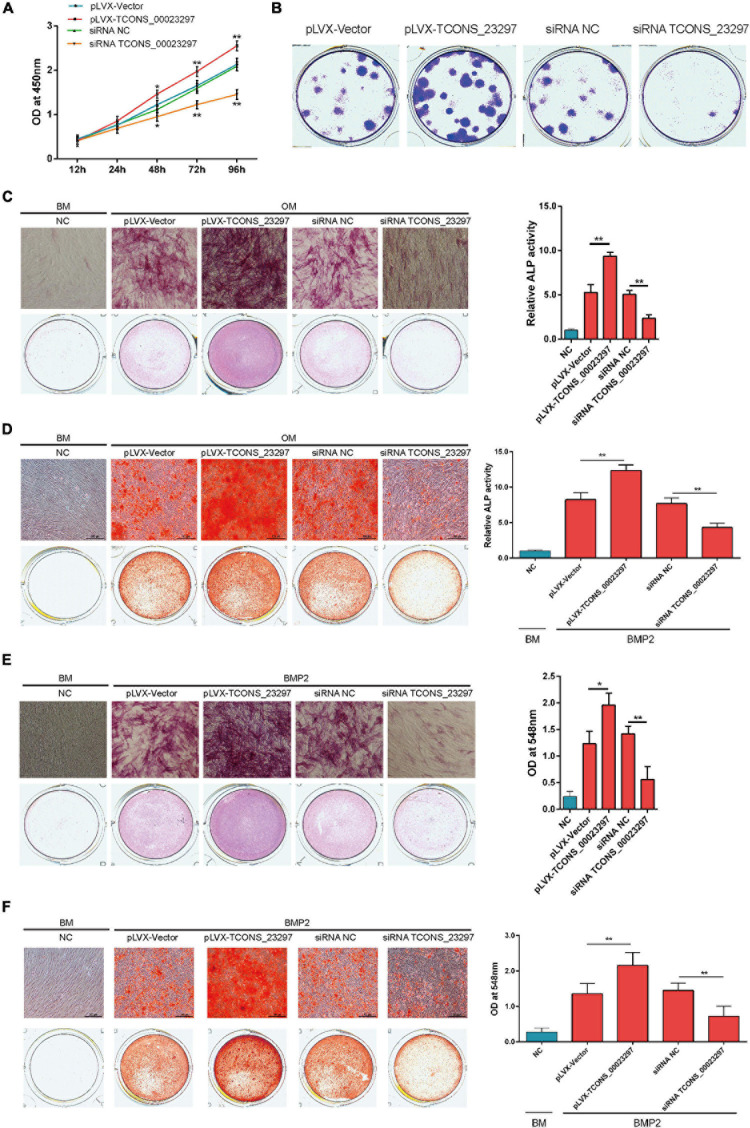
TCONS_00023297 improved the osteogenesis of BMSCs. **(A)** CCK-8 was used to detect the effect of TCONS_00023297 on the proliferation of hBMSCs. NC was indicated negative control and pLVX was indicated overexpression lentiviral vector pLVX. **(B)** Clone proliferation was used to detect the effect of TCONS_00023297 on the proliferation of hBMSCs. **(C)** Alkaline phosphatase staining and quantitative analysis were used to detect the effect of TCONS_00023297 on the osteogenic differentiation of hBMSCs. BM, Basal medium. OM, osteogenic medium. BMP2, Bone morphogenetic protein 2; and ALP, alkaline phosphatase. **(D)** Alizarin red staining and quantitative analysis were used to detect the effect of TCONS_00023297 on the osteogenic differentiation of hBMSCs. **(E)** Alkaline phosphatase staining and quantitative analysis were used to detect the effect of TCONS_00023297 on BMP2-induced osteogenesis of hBMSCs. **(F)** Alizarin red staining and quantitative analysis were used to detect the effect of TCONS_00023297 on BMP2-promoted osteogenesis of hBMSCs. All data are expressed as the means ± standard deviations. “*”means *P* < 0.05, and “**”means *P* < 0.01.

**FIGURE 3:**
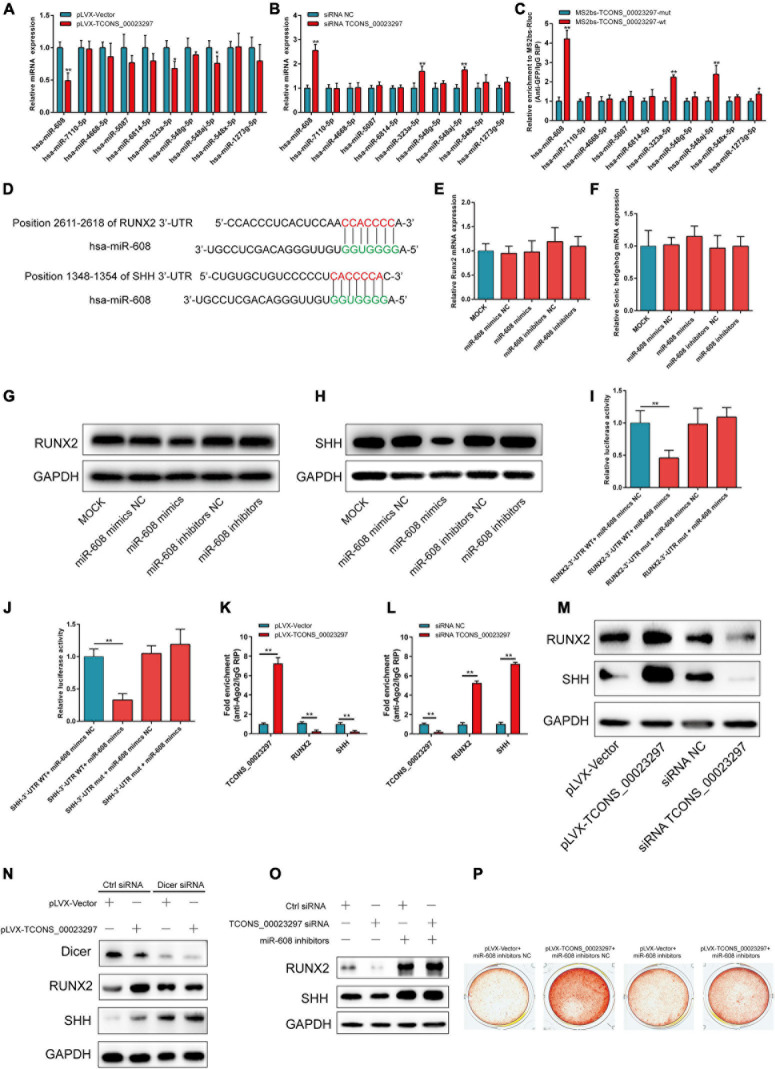
TCONS_00023297 promotes the osteogenesis of BMSCs by regulating miR-608/RUNX2/SHH. **(A)** qRT-PCR was used to detect the effect of TCONS_00023297 overexpression on miRNA expression. **(B)** qRT-PCR was used to detect the effect of TCONS_00023297 knockdown on miRNA expression. **(C)** MS2bs RNA pulldown assay detected miRNAs combined with TCONS_00023297. **(D)** TargetScan bioinformatics software predicted the binding sites of miR-608 and the RUNX2/SHH 3′-UTR. **(E)** qRT-PCR detected the effect of miR-608 on the expression level of RUNX2 mRNA. **(F)** qRT-PCR detected the effect of miR-608 on the expression level of SHH mRNA. **(G)** Western blot analysis of the expression of miR-608 at the RUNX2 protein level. **(H)** Western blot analysis of the expression of miR-608 at the SHH protein level. **(I)** Luciferase reporter gene verification of the combination of miR-608 and RUNX2-3′-UTR. **(J)** Luciferase reporter gene verification of the combination of miR-608 and SHH-3′-UTR. **(K)** Ago2 pulldown detection of TCONS_00023297 overexpression and Ago2 binding. **(L)** Ago2 pulldown assay detected the binding of TCONS_00023297 knockdown and Ago2. **(M)** Western blot analysis was used to detect the effect of TCONS_00023297 on the protein expression of RUNX2 and SHH. **(N)** TCONS_00023297 affected the expression of RUNX2 and SHH protein after Dicer knockdown. **(O)** Effect of TCONS_00023297 on the expression of RUNX2 and SHH protein after miR-608 inhibition. **(P)** Alizarin red staining was used to detect the effect of TCONS_00023297 on the osteogenic differentiation of hBMSCs after miR-608 inhibition. All data are expressed as the means ± standard deviations. “*”means *P* < 0.05, and “**”means *P* < 0.01.

The CCK-8 assays showed that TCONS_00023297 overexpression could promote the proliferation of hBMSCs, and inhibition of TCONS_00023297 could inhibit the proliferation of hBMSCs (Figure 2A). The clone proliferation assay showed that the overexpression of TCONS_00023297 could promote the clone formation of hBMSCs, and the inhibition of TCONS_00023297 could inhibit the clone formation of hBMSCs (Figure 2B). Compared with the control group, the clone size and number of hBMSCs in the TCONS_00023297 overexpression and knockdown groups were significantly different (Figure 2B). On the fifth day of osteogenic differentiation, ALP staining showed that overexpression of TCONS_00023297 could promote the ALP activity of hBMSCs, and inhibiting the expression of TCONS_00023297 could inhibit the ALP activity of hBMSCs. Compared with that in the control group, the ALP activity in the TCONS_00023297 overexpression group and knockdown group were significantly different (Figure 2C). On the 14th day of osteogenic differentiation, alizarin red staining showed that the overexpression of TCONS_00023297 could promote the formation of calcium nodules in hBMSCs, and inhibiting the expression of TCONS_00023297 could inhibit the formation of calcium nodules in hBMSCs (Figure 2D). Compared with the control group, the contents of calcium nodules in the overexpression group and knockdown group of TCONS_00023297 were significantly different (Figure 2D). BMP2 at a final concentration of 0.1 μg/ml was added to induce osteogenic differentiation of hBMSCs. ALP staining was used to detect the expression of TCONS_00023297 on the fifth day of osteogenic differentiation. The overexpression of TCONS_00023297 promoted the ALP activity of hBMSCs, and the inhibition of TCONS_00023297 expression inhibited the ALP activity of hBMSCs (Figure 2E). Compared with the control group, the ALP activity of the TCONS_00023297 overexpression group and knockdown group was significantly different (Figure 2E). BMP-2 at a final concentration of 0.1 μg/ml was added to induce osteogenic differentiation of hBMSCs. Alizarin red staining showed that overexpression of TCONS_00023297 could promote the formation of calcium nodules in the hBMSCs, and inhibition of TCONS_00023297 could inhibit the formation of calcium nodules in the hBMSCs (Figure 2F). The quantitative analysis of the calcium nodules showed that there were significant differences in the contents of calcium nodules between the overexpression group and knockdown group compared with the control group (Figure 2F). Our previous study found that TCONS_00023297 could promote the osteogenic differentiation of hBMSCs. We further analyzed the mechanism of TCONS_00023297. First, we found that TCONS_00023297 could combine with miRNA through bioinformatics analysis. Overexpression of TCONS_00023297 significantly inhibited the expression of hsa-miR-608, hsa-miR-323a-5p and hsa-miR-548aj-5p (Figure 3A). The expression of TCONS_00023297 was inhibited in hBMSCs by using TCONS_00023297-specific siRNA. The results showed that inhibition of TCONS_00023297 could significantly promote the expression of hsa-miR-608, hsa-miR-323a-5p, and hsa-miR-548aj-5p (Figure 3B). According to the analysis results of bioinformatics software, the mutant plasmids of TCONS_00023297 and different miRNA binding regions were constructed. When hsa-miR- 608-, hsa-miR-323a- 5p-, hsa-miR-548aj- 5p-, and TCONS_00023297-specific binding regions were mutated, the MS2b pulldown test was used, and the results showed that the binding of TCONS_00023297-wt to hsa-miR-608, hsa-miR-323a-5p, and hsa-miR-548aj-5p was significantly higher than that of TCONS_00023297-mut (Figure 3C). The above results show that TCONS_00023297 can directly bind to miR-608, hsa-miR-323a-5p, and hsa-miR-548aj-5p, and the binding of miR-608 is more significant. Therefore, we will continue to study the binding relationship between TCONS_00023297 and miR-608 and the regulatory mechanism. Using online biological analysis software, TargetScan analysis found that miR-608 can bind to the RUNX2 and SHH mRNA 3′-UTR regions (Figure 3D). The hBMSCs were transfected with miR-608 mimics and miR-608 inhibitors to regulate the expression of miR-608 in the hBMSCs. mRNA was extracted on the third day after transfection, and the expression of RUNX2 and SHH was detected by qRT-PCR. The test results showed that miR-608 mimics and miR-608 inhibitors had no significant effect on the expressions of RUNX2 and SHH mRNA levels (Figures 3E,F). The hBMSCs were transfected with miR-608 mimics and miR-608 inhibitors to regulate the expression of miR-608 in the hBMSCs. The protein was extracted on the third day after transfection, and the protein expression of RUNX2 and SHH was detected by Western blotting. The test results showed that miR-608 mimics inhibited the protein expressions of RUNX2 and SHH, while miR-608 inhibitors promoted the protein expression of RUNX2 and SHH (Figures 3G,H). Wild-type (RUNX2-3′-UTR-WT) and mutant (RUNX2-3′-UTR-mut) luciferase reporter gene vectors of the miR-608 and RUNX2 binding regions, respectively, and miR-608 mimics NC, miR-608 mimics, miR-608 inhibitors NC, and miR-608 inhibitors were cotransfected with hBMSCs. Luciferase detection was performed on the second day after transfection. The test results showed that miR-608 mimics could significantly inhibit RUNX2-3′-UTR-WT luciferase activity without affecting the luciferase activity of RUNX2-3′-UTR-mut (Figure 3I). Wild-type (SHH-3′-UTR-WT) and mutant (SHH-3′-UTR-mut) luciferase reporter gene vectors of the miR-608 and SHH binding regions, respectively, and miR-608 mimics NC, miR-608 mimics, miR-608 inhibitors NC, and miR-608 inhibitors were cotransfected with hBMSCs. Luciferase detection was performed on the second day after transfection. The test results showed that miR-608 mimics could significantly inhibit SHH-3′-UTR-WT luciferase activity without affecting the luciferase activity of SHH-3′-UTR-mut (Figure 3J). Using an RNA pull-down experiment, it was found that overexpression of TCONS_00023297 could promote the binding of Ago2 and TCONS_00023297 and reduce the binding of Ago2, RUNX2, and SHH (Figure 3K). In contrast, inhibiting the expression of TCONS_00023297 reduced the combination of Ago2 and TCONS_00023297 and increased the combination of Ago2, RUNX2, and SHH (Figure 3L). The above results indicate that TCONS_00023297 competes with RUNX2 and SHH mRNA to bind to Ago2, indicating that TCONS_00023297 regulates the expression of RUNX2 and SHH through the ceRNA mechanism. Since the Dicer enzyme is an important key protein for miRNA production and function, to verify that TCONS_00023297 regulates the expression of miR-608 through the ceRNA mechanism to further regulate the expression of RUNX2 and SHH protein levels, we first used siRNA to reduce the expression of the Dicer enzyme in the hBMSCs and then detected the expression of RUNX2 and SHH protein levels with TCONS_00023297. The test results showed that the overexpression of TCONS_00023297 could promote the expression of RUNX2 and SHH protein levels when the Dicer functions were normal, and when the Dicer enzyme was knocked down by siRNA, the effect of TCONS_00023297 overexpression on the levels of RUNX2 and SHH protein expression was weakened (Figures 3M,N). To further verify that the expression of RUNX2 and SHH protein levels affected by TCONS_00023297 is mediated by miR-608, we used miR-608 inhibitors to inhibit the function of miR-608 in hBMSCs, then reduced the expression of TCONS_00023297, and detected the expression of RUNX2 and SHH protein levels. The test results showed that TCONS_00023297 expression inhibition can reduce the expression of RUNX2 and SHH protein levels when miR-608 functions are normal (Figure 3O). When miR-608 inhibitors were used to inhibit the function of miR-608 in hBMSCs, the effect of TCONS_00023297 on the expression of RUNX2 and SHH protein levels was weakened (Figure 3O). To further verify that the effect of TCONS_00023297 on the osteogenic differentiation of hBMSCs is mediated by miR-608, we used miR-608 inhibitors to inhibit the function of miR-608 in the hBMSCs and then overexpressed TCONS_00023297 to test the osteogenic differentiation function of hBMSCs. The results of Alizarin Red staining showed that the overexpression of TCONS_00023297 can promote the osteogenic differentiation of hBMSCs when miR-608 function is normal. When miR-608 inhibitors were used to inhibit the function of miR-608 in hBMSCs, the effect of TCONS_00023297 on the osteogenic differentiation of hBMSCs was weakened (Figure 3P).

### LncRNA TCONS_00023297 Regulates Adipogenic Differentiation of BMSCs

**FIGURE 4:**
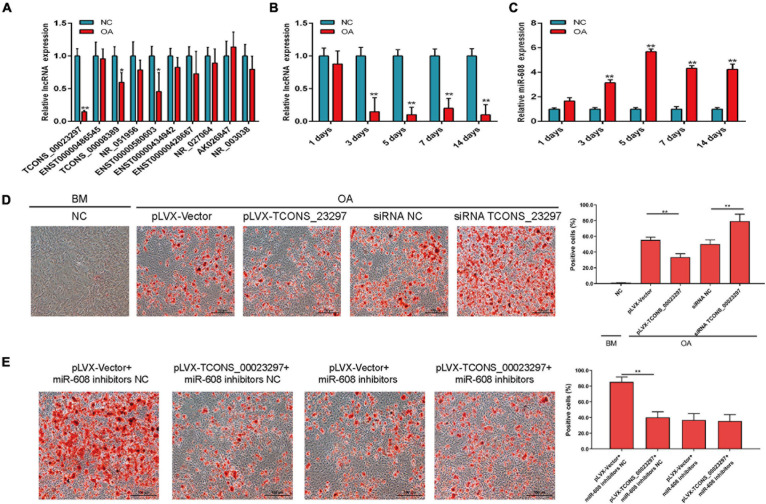
LncRNA TCONS_00023297 regulates adipogenic differentiation of BMSCs. **(A)** Real-time fluorescent quantitative PCR was used to detect the expression of lncRNA on the 3rd day of adipogenic differentiation. NC, negative control; OA, Adipogenic medium. **(B)** TCONS_00023297 expression at different stages of hBMSC adipogenic differentiation induction. **(C)** miR-608 expression at different stages of hBMSC adipogenic differentiation induction. **(D)** Quantitative analysis of the effect of TCONS_00023297 on the adipogenic differentiation of hBMSCs. Scale bar 100 μm. **(E)** Quantitative analysis of the effect of TCONS_00023297 on the adipogenic differentiation of hBMSCs with miR-608 knockdown. Scale bar 100 μm. All data are expressed as the means ± standard deviations. “*”means *P* < 0.05, and “**”means *P* < 0.01.

In the process of osteoporosis, the ability of hBMSCs to differentiate into osteoblasts is weakened, and the ability to differentiate into fats is enhanced. Therefore, we used qRT-PCR to verify the expression changes of lncRNAs on the third day of induction of adipogenic differentiation. The qRT-PCR results showed that lncRNAs were significantly different on the third day of adipogenic differentiation, and TCONS_00023297, TCONS_00008389, and ENST00000580603 were significantly decreased on the third day of adipogenic differentiation (Figure 4A). qRT-PCR detection found that TCONS_00023297 was significantly reduced on the third day of hBMSC adipogenic differentiation. Therefore, we further used qRT-PCR to detect the expression changes of TCONS_00023297 on days 1, 3, 5, 7, and 14 of adipogenic differentiation. The test results showed that the expression of TCONS_00023297 on the third, fifth, seventh, and 14th days of adipogenic differentiation induction was significantly lower than that of the control group (Figure 4B). qRT-PCR was used to detect the expression changes of miR-608 on days 1, 3, 5, 7, and 14 of adipogenic differentiation. The test results showed that the expression of miR-608 was significantly higher than that of the control group on days 3, 5, 7, and 14 of adipogenic differentiation (Figure 4C). After adipogenic differentiation on the 14th day, oil red O staining was used to detect that the overexpression of TCONS_00023297 could inhibit the adipogenic differentiation of hBMSCs, and inhibition of the expression of TCONS_00023297 could promote the adipogenic differentiation of hBMSCs (Figure 4D). To further verify that the effect of TCONS_00023297 on the adipogenic differentiation of hBMSCs is mediated by miR-608, we used miR-608 inhibitors to inhibit the function of miR-608 in hBMSCs and then overexpressed TCONS_00023297 to detect the osteogenic differentiation function of hBMSCs. The results of oil red O staining showed that the overexpression of TCONS_00023297 could inhibit the adipogenic differentiation of hBMSCs when miR-608 functioned normally. When miR-608 inhibitors were used to inhibit the function of miR-608 in hBMSCs, the effect of TCONS_00023297 overexpression on the adipogenic differentiation function of hBMSCs was weakened (Figure 4E).

### LncRNA TCONS_00023297 Regulates the Angiogenesis–Osteogenesis Coupling Process

**FIGURE 5:**
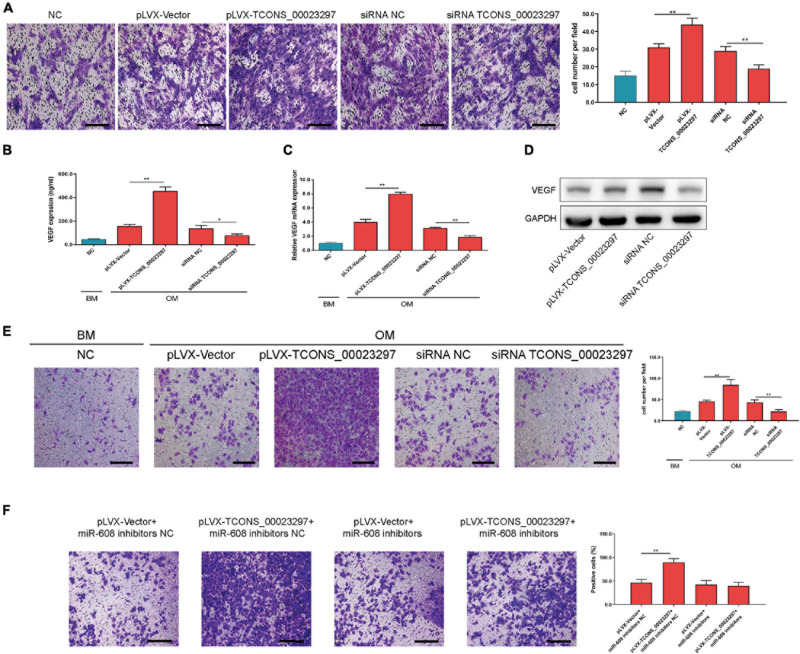
LncRNA TCONS_00023297 regulates the angiogenesis–osteogenesis coupling process. **(A)** Quantitative analysis of the effect of TCONS_00023297 on the migration of hBMSCs. Scale bar 100 μm. **(B)** ELISA detected the expression of VEGF in the hBMSC culture supernatant. **(C)** Real-time fluorescent quantitative PCR detected the expression of VEGF mRNA in hBMSCs. **(D)** Western blot detected the expression of VEGF in hBMSCs. **(E)** Transwell assays were used to detect the effect of TCONS_00023297 on HUVEC migration and with quantitative analysis. Scale bar 100 μm. **(F)** Transwell assays were used to detect the effect of TCONS_00023297 on HUVEC migration after miR-608 knockdown and with quantitative analysis. Scale bar 100 μm. All data are expressed as the means ± standard deviations. “*”means *P* < 0.05, and “**”means *P* < 0.01.

The migration function of hBMSCs has an important influence on the osteogenic differentiation ability of hBMSCs. Using Transwell detection, overexpression of TCONS_00023297 was found to promote the migration of hBMSCs, and inhibition of the expression of TCONS_00023297 could reduce the migration of hBMSCs (Figure 5A). The expression of VEGF has an important influence on the migration and vascularization ability of HUVECs. Using ELISA (Figure 5B), qRT-PCR (Figure 5C), and Western blot (Figure 5D) detection, we found that overexpression of TCONS_00023297 could promote the expression of VEGF in hBMSCs, and inhibition of the expression of TCONS_00023297 could reduce the expression of VEGF in hBMSCs. The culture supernatant was used in the Transwell experiment. Transwell detection found that the overexpression of TCONS_00023297 could promote the migration of HUVECs, and inhibition of the expression of TCONS_00023297 could reduce the migration of HUVECs (Figure 5E). To further verify that the effect of TCONS_00023297 on HUVEC migration was mediated by miR-608, we used miR-608 inhibitors to inhibit the function of miR-608 in hBMSCs and then overexpressed TCONS_00023297. Transwell assays found that TCONS_00023297 overexpression could promote the migration of HUVECs when miR-608 functioned normally. When miR-608 inhibitors were used to inhibit the function of miR-608 in the hBMSCs, the effect of TCONS_00023297 overexpression on the migration function of HUVECs was weakened (Figure 5F).

### LncRNA TCONS_00023297 Regulates the Osteogenesis *in vivo*

**FIGURE 6:**
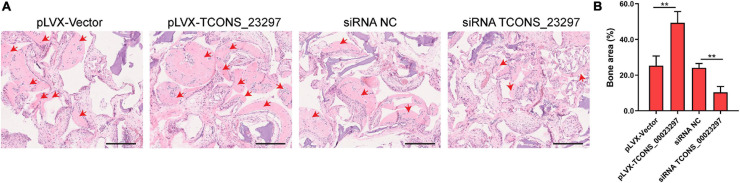
LncRNA TCONS_00023297 regulates osteogenesis *in vivo*. **(A)** H&E staining was used to detect new bone formation. The area indicated by the red arrow was new bone. Scale bar 200 μm **(B)** Quantitative analysis of new bone formation. “**”means *P* < 0.01.

TCONS_00023297 lentiviral overexpression vector and siRNA were used to regulate the expression of TCONS_00023297 in the hBMSCs and were implanted subcutaneously in nude mice, and HE staining was performed 8 weeks after implantation. The overexpression of TCONS_00023297 could promote the formation of ectopic bone under the skin of the hBMSCs and inhibit the expression of TCONS_00023297, which could inhibit the formation of ectopic bone under the skin of the hBMSCs (Figure 6).

## Discussion

Long noncoding RNAs play an important role in maintaining the multidirectional potential of stem cells (Ng et al., 2012). Our systematic functional experiment showed that the knockdown of 147 lncRNAs in mouse embryonic stem cells (ESCs) resulted in changes in gene expression profiles, which was consistent with the knockdown of key pluripotency marker molecules, such as Nanog or Oct4, indicating that lncRNAs are important for maintaining the stability of stem cells (Ng et al., 2012). In addition, lncRNAs can promote and retain the essence of the cell after stem cell differentiation. A total of 6671 lncRNA transcripts were screened from human ESCs by microarray. Further identification revealed that 934 noncoding RNAs were involved in the differentiation of ESCs into neural precursor cells *in vitro* (Rybak et al., 2008; Le et al., 2009). LncRNAs play an important role in regulating the cell fate and function. In many cases, abnormal expression of lncRNAs is related to diseases. In terms of chondrogenesis and osteogenesis, there are still few studies on how lncRNAs regulate the differentiation of stem cells or precursor cells toward chondrocytes or osteoblasts. We speculate that in the next few years, studies on lncRNAs in bone diseases will increase greatly, which will provide us with more relevant data on cartilage and bone development regulation. At present, there is no information on how lncRNAs control the homeostasis of mature cartilage or bone. Such findings will have a profound impact on our understanding of bone diseases, such as OA or osteoporosis, and further determine how we can develop new treatment strategies for these diseases. In this study, we examined the expression of lncRNAs during osteogenic and adipogenic differentiation of BMSCs. Using lncRNA microarray and qRT-PCR, we found that TCONS_00023297 could regulate the osteogenic and adipogenic differentiation of BMSCs.

miRNA play an important role in osteogenesis (Zhuo et al., 2020). MiR-608 is a newly discovered miRNA that can be detected in a variety of tissues and cells. It has been reported that the expression of miR-608 is significantly down-regulated in liver cancer, lung cancer, rectal cancer and glioma. It can inhibit the proliferation, invasion and metastasis of tumor cells, promote the apoptosis of tumor cells, and inhibit the occurrence and development of tumors. As of now, no studies have reported the role of miR-608 in the pathogenesis of osteoporosis. Our results show that miR-608 can inhibit the expression of Runx2 and Shh in bone marrow mesenchymal stem cells and inhibit the osteogenic differentiation of BMSCs. It has been reported that miR-608 could inhibit Runx2 expression by inhibiting the PI3K/Akt signaling pathway (Chen et al., 2019). Therefore, miR-608 may be a new target for the treatment of osteoporosis.

In recent years, the coupling relationship between angiogenesis and bone formation has become a new breakthrough point in the treatment of osteoporosis. The maintenance of normal bone formation requires mesenchymal stem cells to continuously differentiate into bone progenitor cells and then form osteoblasts to participate in bone formation and maintain bone homeostasis, which is essential for the regeneration and repair of new bone formation and fracture healing, and the whole process requires the participation of blood vessels. Currently, the treatment of osteoporosis is still focused on the regulation of bone resorption and bone formation. The coupled relationship between blood vessels and bone formation can provide a new breakthrough for the treatment of osteoporosis. With the aging of experimental mice, the number of EPCs and osteoblasts decreased, which suggested that the decrease in EPCs may be related to senile osteoporosis [24646994]. In our study, we found that lncRNA TCONS_00023297 could regulate the coupling process of osteogenesis and angiogenesis.

## Conclusion

The expression of TCONS_00023297 increases during osteogenic differentiation; overexpression of TCONS_00023297 promotes the osteogenic differentiation of hBMSCs; the regulation of TCONS_00023297 expression by BMP2 is concentration- and time-dependent; mechanical stimulation can promote the expression of TCONS_00023297; TCONS_00023297 regulates the expression of miR-608 through the ceRNA mechanism; TCONS_00023297 inhibits hBMSCs into adipogenic differentiation; and TCONS_00023297 promotes hBMSCs to secrete VEGF. TCONS_00023297 regulates the osteogenic differentiation, adipogenic differentiation, and osteogenesis–angiogenesis coupling of hBMSCs through the TCONS_00023297/miR-608/RUNX2/SHH signaling axis.

## Data Availability Statement

The original contributions presented in the study are included in the article, further inquiries can be directed to the corresponding author/s.

## Ethics Statement

The studies involving human participants were reviewed and approved by Ethics Committee of First Affiliated Hospital of Zhengzhou University. The patients/participants provided their written informed consent to participate in this study.

## Author Contributions

HW and LY: conception and design. PW, YL, and HW: experiments and/or data analysis. YZ: intellectual input and supervision. LY and HW: article writing. All authors contributed to the article and approved the submitted version.

## Conflict of Interest

The authors declare that the research was conducted in the absence of any commercial or financial relationships that could be construed as a potential conflict of interest.
